# Necrotizing pelvic infection after rectal resection. A rare indication of endoscopic vacuum-assisted closure therapy. A case report

**DOI:** 10.1016/j.ijscr.2019.06.054

**Published:** 2019-06-27

**Authors:** Tomáš Řezáč, Martin Stašek, Pavel Zbořil, Katherine Vomáčková, Linda Bébarová, Jan Hanuliak, Čestmír Neoral

**Affiliations:** a Department of Surgery I, University Hospital Olomouc, Olomouc 77900, Czech Republic; b Department of Surgery I, Faculty of Medicine and Dentistry, Palacky University Olomouc, Olomouc 77900, Czech Republic

**Keywords:** BMI, Body Mass Index, CT, computed tomography, EVAC, endoscopic vacuum-assisted closure, ASA score, The American Society of Anesthesiologists score, ECOG performance status, The Eastern Cooperative Oncology Group performance status, CR POSSUM, Colorectal Physiological and Operative Severity Score for the Enumeration of Mortality and Morbidity, NPI, necrotizing pelvic infection, ICU, Intensive Care Unit, CRP, C-reactive protein, Low anterior rectal resection, Anastomotic leak in colorectal surgery, Necrotizing pelvic infection, Endoscopic vacuum-assisted closure (EVAC), Case report

## Abstract

**Introduction:**

Anastomotic leak after colorectal surgery is a major problem associated with higher morbidity and mortality. In most cases of contained leaks, treatment recommendations are clear and effective. However, in rare cases like necrotizing pelvic infection, there is no clear treatment of choice, despite the mortality rate almost 21%. We present successful management with endoscopic vacuum-assisted closure therapy.

**The presentation of a case:**

A 68-year-old female patient with BMI 26, hypothyroidism and high blood pressure was indicated to low anterior rectal resection because of high-risk neoplasia of lateral spreading tumor type of the upper rectum. Four days after the primary operation, sepsis (SOFA 12) with diffuse peritonitis and unconfirmed leak according to CT led to surgical revision with loop ileostomy. On postoperative days 6–10, swelling, inflammation and subsequent necrosis of the right groin and femoral region communicating with the leak cavity developed. The endoscopy confirmed a leak of 30% of the anastomotic circumference with the indication of debridement and endoscopic vacuum-assisted closure therapy. EVAC sessions with 3–4 day intervals healed the leak cavity. Secondary healing of the skin defects required 4 months.

**Conclusion:**

Necrotizing pelvic infection after a leak of the colorectal anastomosis is a very rare complication with high morbidity and mortality. Endoscopic vacuum-assisted closure therapy should be implemented in the multimodal therapeutic strategy in case of major leaks, affecting up to 270° of the anastomotic circumference.

## Highlights

• Necrotizing pelvic infection after rectal resection is a rare complication.
• There are no clear recommendations for the treatment of choice.
• Multimodality therapy requires intensive care, antibiotics and usually repeated surgeries.
• Endoscopic vacuum-assisted closure therapy may decrease the disease burden in properly indicated cases.

## 1 Introduction

Anastomotic leak after colorectal resection is a dreaded complication with the incidence ranging from 6% in high volume centers to almost 22% [1]. Anastomotic leak is an important prognostic and predictive factor for early morbidity and mortality and for higher tumor recurrence. Common risk factors include age, male sex, smoking, coronary heart disease, obesity, difficult anastomosis [2] and vascular compromise. Early recognition of the anastomotic leak is important for successful therapy. Rare complications, including necrotic pelvic infection, are still lacking strong recommendations for management and the evidence and experience is limited. Necrotic pelvic infection is very rare with an incidence of 0,02% of admitted patients [3]. The incidence is probably higher but underreported due to poor prognosis and outcomes. Multimodal comprehensive treatment including adequate intensive care, surgical and endoscopic therapy (endoscopic vacuum-assisted closure - EVAC) may change the complication burden and prognosis. The work has been reported in line with the SCARE criteria [4].

## 2 Patient information

The presented case is a 68-year-old female patient with BMI 26, ASA score II, ECOG performance status 0, CR POSSUM operative severity score 6 with mortality prediction 1%, with hypothyroidism and high blood pressure, non-smoker, with a history of Billroth II gastric resection, cholecystectomy and choledochoduodenostomy. The patient exhibited suspected neoplastic lesion of the liver with negative CT guided biopsy. The workup included gastroscopy with chronic gastritis and subsequent colonoscopy with extensive benign polyp of lateral spreading tumor type 15 cm from the anal verge with size 30 × 25 × 25 mm and non-lifting phenomenon contraindicated for endoscopic treatment. A low anterior resection was carried out. The anastomosis was performed with a circular stapler approximately 10 cm from the anal verge. The only peroperative complication was bleeding from sacral excavation with a blood loss of approximately 800 ml. On the fourth postoperative day, septic shock with CT-confirmed inflammatory changes in small pelvis and peritoneal cavity effusion without a confirmed leak. Skin inflammation of the right groin and femoral region developed. Broad-spectrum antibiotics and surgical revision under general anesthesia with abdominal washing and diverting ileostomy was performed. Further intensive care required broad-spectrum antibiotics, the circulation was supported with small doses of norepinephrine. In the following course, general and local clinical conditions were still worsening with localized skin necrosis and maximum SOFA score 12 (Respiratory system +2, Nervous system +3, Cardiovascular system +3, Liver 0, Coagulation +3, Kidneys +1) with 80% mortality risk.

**Fig. 1:**
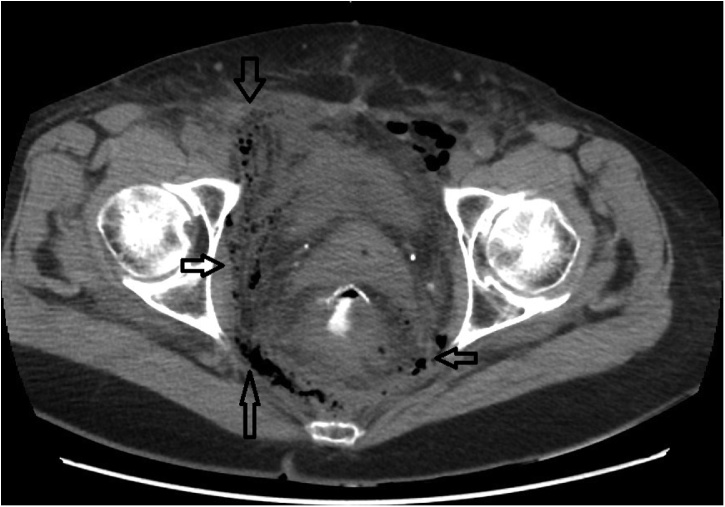
CT scan of an anastomotic leak and infection of the soft tissue in the right lower abdomen.

**Fig. 2:**
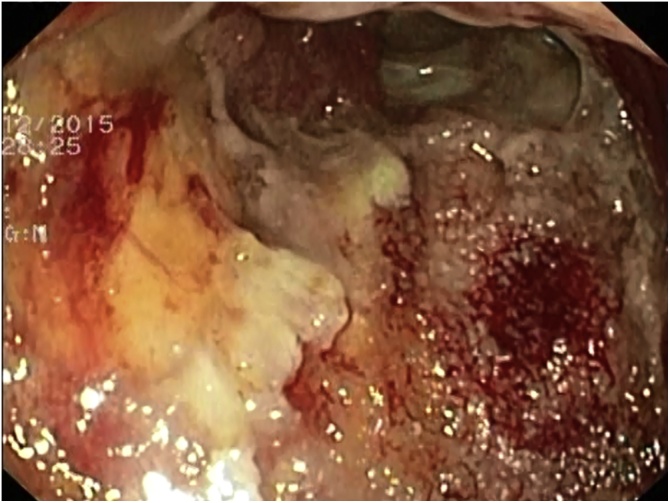
Granulating tissue after the first session of EVAC.

**Fig. 3:**
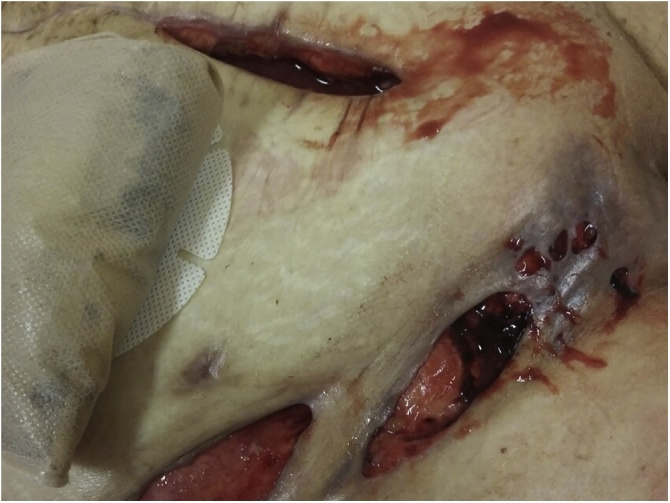
Skin necrosis of the right groin and femoral region.

On the 8th postoperative day, severe necrotic tissue infection in the right groin and a presacral anastomotic leak were confirmed (Fig. 1). Endoscopy showed a 3 cm wide anastomotic dehiscence on the ventrolateral margin with a 5 cm long cavity and pelvic necrotic tissue (Fig. 2). Necrectomy of necrotic skin and soft tissue extending to right lower pelvis was carried out and drained with subsequently proven Enterococcus and Pseudomonas infection (Fig. 3). The leak was managed by EVAC with an extraluminal installation.

The technique consisted of sigmoidoscopic determination of the leak, measuring of the cavity, lavage and necrectomy and application of the foam using overtube. The negative pressure was set to 50–75 mmHg, subsequently to 100–125 mmHg. In the course of EVAC therapy, clinical status was rapidly improved. A total of 6 procedures were performed until there was a defect of 1 cm in diameter. The whole hospital stay took 40 days, the ICU stay 16 days.

**Fig. 4:**
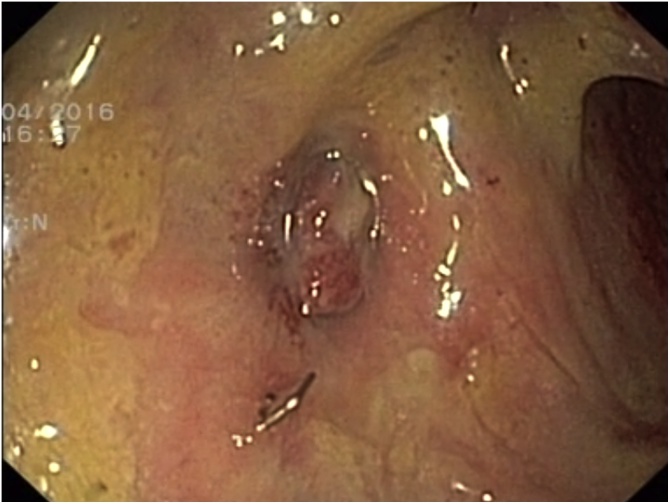
Healed defect 3 months after the termination of EVAC therapy.

Colonoscopy 3 months after surgery showed a healed anastomosis without remanent cavity, fistula or stenosis (Fig. 4). The skin defects were healed after 117 days, the ileostomy was restored 14 months after primary surgery. Within 3 years of follow-up, no rectal fistula, stenosis or continence impairment occurred. The liver lesion disappeared, thus suspected to be caused by ascending biliary infection following choledochoduodenostomy.

## 3 Discussion

Necrotizing pelvic infection (NPI) after rectal resection is a rare but life-threatening complication. The most common cause is perianal or ischiorectal abscess and hemorrhoidectomy, less common causes include rectal carcinoma, inflammatory bowel disease, blunt trauma, perforated diverticulitis, rectal biopsy, sphincter dilatation, and colorectal anastomotic leakage. [5]

NPI is a condition characterized by septic shock and rapidly progressing bacterial infection involving the subcutaneous fascia and deep pelvic fascias, usually sparing muscles in the perianal and perineal region. Based on the anatomy of fascial planes, the infection can spread from the rectum to the presacral space, retrovesical space, perirectal tissue and retroperitoneum or superiorly along Scarpa´s fascia to the anterior abdominal wall. [6]

General clinical signs are usually non-specific, the patient suffers from pain, fever, and weakness. In case of intact skin and deeply spreading infection, paresthesia may occur as a sign of advanced finding as a result of cutaneous nerve damage. Subsequent skin necrosis with crepitation and typical anaerobic smell and septic shock usually progress rapidly to multiple organ failure. [7] Most patients without appropriate treatment died within 48 h from the presentation [8].

The diagnostic workup includes blood markers (blood count, CRP, procalcitonin), CT scan with water-soluble contrast enema and digital rectal examination with possible subsequent endoscopy. CT scan with water-soluble contrast enema shows 100% sensitivity and 96% specificity for anastomotic leak. The main outcomes comprise the extent of leak and leak cavity and the presence of intraabdominal collections and peritonitis non-related to the leak site. Endoscopy shows the exact morphology and localization of the leak, measuring of the cavity and viability of the bowel. The most common infectious agents of synergistic infections are Streptococci with Staphylococcus aureus, coliforms or Bacteroides spp. [9].

The prognosis depends on multiple factors, including age over 60, vascular disease, poor nutritional status, and diagnostic and therapeutic delay. The severity of the sepsis and clinical status is expressed by SOFA Score. The mortality rate differs in case of initial score or the highest score. SOFA score 10–11 means 50% (initial) and 45,8% (highest) mortality, score 12–14 95,2% (initial) and 80% (highest), respectively [10]. Other severity-of-disease classification systems for mortality risk prediction are APACHE score, CR POSSUM serves for colorectal surgery.

Score for necrotizing soft tissue infection – LRINEC (Laboratory Risk Indicator for Necrotizing Fasciitis) - should be used with caution. It has demonstrated a high false negative rate in cases of confirmed necrotizing fasciitis with recommendations to rely on clinical signs of skin necrosis, and subcutaneous gas on imaging studies [11].

Therapy includes complex intensive care including broad spectrum antibiotics, parenteral nutrition, and inotropic support, in case of accessibility, hyperbaric oxygen therapy could be considered. Operative procedures are reserved for patients with frank purulent or feculent peritonitis and unstable vital signs and vary from loop ileostomy with lavage and drainage to resection of the anastomosis and closure of the rectal stump with a terminal colostomy, especially in case of intraperitoneal anastomosis. Revisional surgery for the control of sepsis is rarely necessary if prior diverting ostomy is present, unlike those with extraperitoneal anastomoses. If surgical revision of the abdominal cavity is not mandatory (contained pelvic leak, previous ostomy, the absence of peritonitis signs) or in case of combined surgical approach, the condition may benefit from additional transanal or percutaneous drainage, EVAC therapy, endoscopic stenting or clipping.

EVAC is a method suitable for grade A and B anastomotic leaks up to 270° of the anastomotic circumference. EVAC continuously removes secretions, improves microcirculation, induces granulation and allows the drainage of both the cavity and the rectum. Main contraindications for EVAC include separation of the anastomosis (grade C of anastomotic leakage) [12], visible major vessels in the cavity. Higher anastomoses can make placement of the sponge difficult. EVAC is an accessible, well-tolerated method, usually not requiring general anesthesia.

Endoscopic stenting with covered metal, plastic, and biodegradable stents have also been utilized with success. A stent can be placed only across an end to end anastomosis and the distal end should be 5 or more cm above the anal verge. The low stent placement in the inferior rectal anastomoses may cause rectal pain, tenesmus or fecal incontinence. Stents are suitable for smaller leaks after surgical peritoneal lavage and drainage or in combination with percutaneous drainage [13].

Endoscopic clipping is another possibility for the closure of limited leaks. Through-the-scope clips have a low closure force and are limited in size and usage in a fibrotic and postirradiation field. The over-the-scope clip system (OTSC^®^ System) with larger jaw and increased compression allows full-thickness closure. Commonly, the indication of clip closure is in defects less than 1,5 cm in size and the absence of a pelvic collection. There is only limited evidence of closure of up to 30 mm defects [14] or the application of multiple OTSC clips [15].

Chopra proposed an algorithm for endoscopic treatment prior to the wider implementation of OTSC and EVAC. For patients with defects greater than 2 cm, a diverting ileostomy with EVAC therapy is preferred. For defects less than 2 cm in size in the middle rectum, endoscopic stenting is the treatment of choice. Fibrin sealant is utilized for defects smaller than 3 mm without abscess. For those with abscess, percutaneous drainage is preferred [16]. The general principles of the tailored approach should be implemented, aimed at minimalization of the extent of the intervention, shortening of the procedure and appropriate timing.

Skin necrosis joined with necrotizing infection requires extensive surgical debridement in general anesthesia with subsequent wound healing using wet therapy or vacuum-assisted closure.

## 4 Conclusion

Anastomotic leak is a feared complication of colorectal resections, causing higher morbidity, mortality and tumor recurrence. The combination with necrotizing pelvic infection is highly lethal and causes a significant prolongation of the therapy and the decrease of the quality of life. Early diagnosis is mandatory for good therapeutic results. The intensive care, antibiotic therapy, surgical, endoscopic and local therapy including aggressive surgical debridement are necessary parts of the management. EVAC is a well-known modality in anastomotic leak treatment with increasing indications. Necrotizing pelvic infection might be a new indication for EVAC not reported to date. In case of appropriate timing and adequate management, EVAC could contribute to the preservation of the anastomosis and reduce invasivity. Further evidence is necessary.

## Sources of funding

Supported by University Hospital Olomouc, Czech Republic.

## Ethical approval

For case report our Institute exempted to take ethical approval.

## Consent

Informed consent for the publication of this work has been given by the patient.

## Author contribution

MUDr. Tomáš Řezáč. - Methodology, resources, writing.

MUDr. Martin Stašek, PhD. - methodology, resources, review & editing supervision.

MUDr Pavel Zbořil, Ph.D. - review and editing.

MUDr. Katherine Vomáčková, PhD. - Proof reading, language correction, review and editing supervision.

MUDr. Linda Bébarová. Review and editing.

MUDr. Jan Hanuliak, - resources, review.

Prof. MUDr. Čestmír Neoral, CSc. – review and editing.

## Registration of research studies

The case report is not a part of a clinical trial.

## Guarantor

MUDr. Tomáš Řezáč.

MUDr. Martin Stašek, PhD.

## Provenance and peer review

Not commissioned, externally peer-reviewed.

## Statement on confidential information

All the figures used in this paper were checked and they do not contain any confidential patient details including the patient's name or the institution.
